# New Onset of Symptomatic Peptic Ulcer Disease Postpartum Secondary to Nonsteroidal Anti-Inflammatory Drug Use

**DOI:** 10.1155/2024/6422824

**Published:** 2024-06-11

**Authors:** Maggie Tallmadge, Margaret MacBeth, Anna Palatnik

**Affiliations:** Department of Obstetrics and Gynecology Medical College of Wisconsin, Milwaukee, Wisconsin, USA

**Keywords:** cesarean section, NSAID, peptic ulcer, postoperative pain, postpartum care

## Abstract

The use of nonsteroidal anti-inflammatory drug (NSAID) medications is a risk factor for peptic ulcer disease (PUD). PUD in the postpartum period is rare, despite the common use of NSAIDs. A G1P0 presented 6 days postcesarean section with fatigue, lightheadedness, melenic stools, and a hemoglobin of 5.4 g/dL after using NSAIDs and acetaminophen for postoperative pain control. An esophagogastroduodenoscopy (EGD) was performed for a suspected upper gastrointestinal bleed and found one gastric and one duodenal ulcer. Though typically used for a short course in the postpartum period, NSAIDs remain a predisposing risk factor for PUD postpartum, and patients and providers must be aware of this risk.

## 1. Introduction

Upper gastrointestinal bleeding (UGIB) is rare in the postpartum period and had seldom been reported in the literature [1–3]. Peptic ulcer disease (PUD), though rare, is an etiology for UGIB, and nonsteroidal anti-inflammatory drugs (NSAIDs) can also contribute [1, 4]. We present a case of UGIB due to PUD developing postpartum secondary to NSAID use for postcesarean pain control.

## 2. Case Report

A 27-year-old woman with G1P0 presented for postdate induction of labor at 41 weeks and 4 days. Her pregnancy was complicated by iron deficiency anemia. The history of exposure to tobacco, alcohol, or illicit drug use was negative. She did not have nausea or vomiting during pregnancy. During her third trimester, she had complained of mild gastroesophageal reflux but did not require medication for relief. She had no prior history of gastrointestinal bleeding or PUD. On admission for induction, her hemoglobin was 11.5 g/dL. She received oxytocin for induction. Amniotomy was performed with clear amniotic fluid. Throughout the induction, electronic fetal monitoring was intermittently Category 2 with spontaneous 1–2-min decelerations with nadir to 80–90 bpm but with moderate variability throughout and reactive in between. She progressed to complete cervical dilation and started pushing at +2 fetal station. She pushed over one contraction and electronic fetal monitoring demonstrated bradycardia down to 60 bpm. Position changes and fluid resuscitation were attempted without resolution of the fetal bradycardia. The decision was made to proceed with emergent primary low transverse cesarean section. General anesthesia was required as epidural coverage was not sufficient. The patient received cefazolin and azithromycin for antimicrobial prophylaxis. There were no intraoperative complications.

The inpatient postoperative course was uncomplicated. For postoperative pain control, the patient received 1 g acetaminophen every 6 h and 600 mg ibuprofen every 6 h, with 5 mg oxycodone every 4 h if needed for breakthrough pain. The patient was discharged home on postoperative Day 3. On postoperative Day 6, the patient called the clinic reporting three episodes of black stools with associated lightheadedness, weakness, fatigue, and dyspnea on exertion. Postpartum bleeding was appropriate, and the incision was healing well. For pain control at home, the patient was taking 600 mg ibuprofen every 6 h alternating with 1 g acetaminophen every 6 h. She was directed to the emergency department for further evaluation and management.

**Figure 1:**
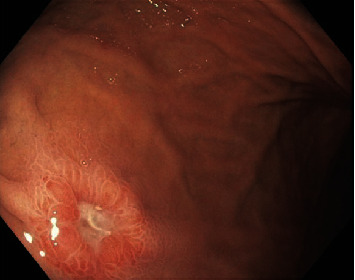
Five-millimeter gastric body ulcer without active bleeding.

**Figure 2:**
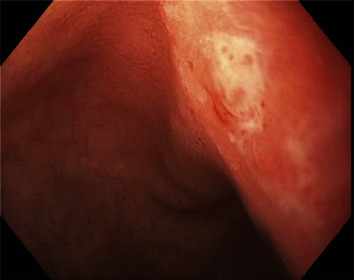
Three-millimeter duodenal ulcer with a clean base and no active bleeding.

In the emergency department, laboratory work-up demonstrated severe anemia with hemoglobin of 5.4 g/dL, blood urea nitrogen of 29 mg/dL, and blood urea nitrogen: creatinine ratio of 50. Rectal exam was guaiac positive. An esophagogastroduodenoscopy (EGD) was performed for suspected UGIB the following day and revealed a 5-mm gastric body ulcer with a flat pigmented spot without active bleeding and a 3-mm duodenal ulcer with a clean, white base without active bleeding (Figures 1 and 2). Biopsies were taken for *H. pylori*, which were negative.

Upon hospital admission, three units of packed red blood cells were transfused given the profound anemia. Intravenous proton pump inhibitor, pantoprazole, was started given the presumed diagnosis of UGIB. Given the appropriate response to transfusion and hemodynamic stability, the patient was discharged home on hospital Day 3 of readmission and instructed to discontinue use of all NSAIDs, to continue 40 mg oral pantoprazole twice daily for 8 weeks, and to receive once daily 200 mg iron infusions for 3 days.

The patient underwent surveillance endoscopy 10 weeks after hospital discharge to ensure ulcer healing. Bleeding did not recur, and follow-up EGD was normal.

The subject of this report gave informed consent and patient anonymity was preserved. A written consent has been obtained from the patients, but there are no patient-identifiable data included in this case report.

## 3. Discussion

This case describes UGIB in the postpartum period presumably due to NSAID usage for postcesarean pain control. The UGIB is defined as a bleed in the gastrointestinal tract that occurs proximal to the Ligament of Treitz. While the annual incidence rate of hospitalization for UGIB is 65 per 100,000 individuals in the United States, this event is rare in the postpartum period and has rarely been reported in the literature [1–3]. There are various etiologies of UGIB, none of which is unique to pregnancy or the postpartum period. Common causes include gastric, duodenal, and small bowel erosions/ulcerations secondary to *H. pylori*; use of NSAIDs; alcohol; esophageal and gastric varices; angiodysplasia; mass lesions; and traumatic causes, including the Mallory-Weiss tears [1]. Nausea and vomiting, common during pregnancy and labor, can contribute to the Mallory-Weiss tears and mucosal tears at the gastroesophageal junction that can cause profuse bleeding and hemodynamic instability. In the literature, there has been a report of a postpartum UGIB secondary to a Mallory-Weiss tear caused by retching and vomiting during labor, which was ultimately treated with an endoscopic procedure in the immediate postpartum period [5]. However, postpartum UGIB due to PUD is rare. In the case we described, the patient had only one risk factor for UGIB, and it was consistent postpartum use of NSAIDs following cesarean delivery.

While NSAIDs are routinely used during the postpartum period for pain control, due to their short-term use, the risk of NSAID-related complications and the presence of new PUD are low [6]. In addition, the incidence of PUD itself is infrequent, and the estimated incidence in the general population is approximately 0.1%–0.3% per year, declining rapidly over the past few decades [4]. Given its scarcity in the general population, PUD has seldom been reported in the postpartum population. There have been few cases reported of perforated peptic ulcers in the postpartum population, all following cesarean sections [7]. There appears to be an associated risk of postpartum PUD with cesarean section versus vaginal delivery. This could be multifactorial. Pain is often more severe following major abdominal surgery, leading to greater usage of NSAIDs and, therefore, increased risk of ulcer formation. In addition, adequate blood flow to the gastrointestinal tract is an important protective factor against PUD. One characteristic of NSAIDs that leads to the creation of ulcers includes their ability to reduce the blood flow to the stomach that usually helps both wash away gastric acid and repair mucosal injury [8]. One could theorize that the hemodynamic changes occurring during the cesarian section, compounded by NSAID use, could contribute to the lack of blood flow and the creation of peptic ulcers as seen in our patient.

It is critical to control pain in the postpartum period as uncontrolled pain can ultimately hinder the ability of a woman to care for herself and her new baby [9]. Therefore, a stepwise, multimodal approach that utilizes both pharmacologic and nonpharmacologic modalities is commonly used to address pain after cesarean delivery [9]. Per guidance from the American College of Obstetricians and Gynecologists, initial pharmacologic choices include acetaminophen and NSAIDs with the subsequent addition of opioids for additional pain [9]. While NSAIDs are prescribed routinely for postpartum pain, they do carry side effects that one must consider, although their risk of significant acute UGIB in the postpartum period appears low given the low-dose and short-term use in overall healthy individuals. It is prudent that obstetric providers consider their patient's risk factors for UGIB, including a “history of PUD; heart disease; and coprescription of antiplatelets, corticosteroids, and anticoagulants” before recommending or prescribing NSAIDs [6]. If the patient has any history of peptic ulcers or UGIB, it may be beneficial to prescribe daily proton pump inhibitors to reduce her risk of UGIB in the postpartum period [6]. This is particularly true in those taking NSAIDs scheduled for more severe pain after the cesarian section. As evidenced by our case report, even patients without risk factors can experience an UGIB postpartum due to the use of NSAIDs, and both patients and providers must be aware of this potential complication.

## 4. Conclusion

In conclusion, effective pain control in the postpartum period, especially following a cesarean delivery, is essential for the well-being of the mother and her ability to care for her newborn. A stepwise, multimodal approach incorporating both pharmacologic and nonpharmacologic methods is recommended, but careful consideration of individual risk factors is crucial to avoid complications such as UGIB associated with NSAID use.

## Data Availability

This is a case report, and all data is included with the submission.
